# High Sensitivity Surface Plasmon Resonance Sensor Based on a Ge-Doped Defect and D-Shaped Microstructured Optical Fiber

**DOI:** 10.3390/s22093220

**Published:** 2022-04-22

**Authors:** Nilson H. O. Cunha, José P. da Silva

**Affiliations:** Post-Graduated Program in Electrical and Computer Engineering, Technology Center, Federal University of Rio Grande do Norte, Natal 59078-970, Brazil; jose.patrocinio@ufrn.br

**Keywords:** optical sensors, microstructured optical fiber, surface plasmon resonance, refractive index detection, Ge-doped defect

## Abstract

In this work a plasmonic sensor with a D-Shaped microstructured optical fiber (MOF) is proposed to detect a wide range of analyte refractive index $\left(\mathrm{RI}\;;n_{a}\right)$ by doping the pure silica $\left({\mathrm{SiO}}_{2}\right)$ core with distinct concentrations of Germanium Dioxide $\left({\mathrm{GeO}}_{2}\right)$, causing the presentation of high spectral sensitivity. In this case, the fiber is shaped by polishing a coating of ${\mathrm{SiO}}_{2}$, on the region that will be doped with ${\mathrm{GeO}}_{2}$, in the polished area, a thin gold (Au) layer, which constitutes the plasmonic material, is introduced, followed by the analyte, in a way which the gold layer is deposited between the ${\mathrm{SiO}}_{2}$. and the analyte. The numerical results obtained in the study shows that the sensor can determine efficiently a range of 0.13 refractive index units (RIU), with a limit operation where $n_{a}$ varies from 1.32 to 1.45. Within this application, the sensor has reached an average wavelength sensitivity (WS) of up to 11,650.63 nm/RIU. With this level of sensitivity, the D-Shaped format and wide range of $n_{a}$ detection, the proposed fiber has great potential for sensing applications in several areas.

## 1. Introduction

Optical sensors, in general, have the function of determining characteristics of unknown materials, be it temperature, pressure, color, distance or any other parameter. These devices can be constructed from conventional fibers, D-shaped, H-shaped, or any other shape fibers, assuming that it complies with the principle of operation, which is the emission and reception of light and the interpretation of the data received. However, these types of sensors can be optimized with compatible techniques, as is the case of the optical sensors that use the phenomenon of surface plasmon resonance (SPR).

In addition, the SPR-based sensors have been widely studied in recent years, mainly due to their real-time response, greater accuracy, sensitivity, adaptability and ease in construction [1,2,3,4], which make these types of sensors very attractive in various applications.

The Effect of SPR occurs when light excites an interface between two materials, specifically a metal–dielectric interface, which will cause oscillations of charge density along the interface, these oscillations are called surface plasmon oscillations (SPO) and the quantum of these oscillations is called surface plasmon mode (SPM) [3,4]. The surface plasmons are evanescent waves, that is, they are accompanied by a longitudinal electric field that decays exponentially along the propagation, therefore, the visualization and use of the SPR effect is performed locally, hence the term localized surface plasmon resonance (LSPR) [2,3,4,5,6,7,8]. In this work, the SPR effect is stimulated in the region between the gold layer and the analyte, when the structure is excited at optical frequencies.

SPR-based sensors can be easily obtained. In most cases its construction is based only by introducing a metal layer in contact with an excited dielectric material at optical frequencies. However, some models of plasmonic sensors are obtained from special optical fibers, such as those designed from Photonic Crystal Fibers (PCF). In this case, the distribution of the air holes that characterize the PCF can increase the precision of the device, however, they can be more difficult to build. PCF present periodic distribution of air holes along the direction of signal propagation. On the other hand, Microstructured Optical Fibers (MOF) can present a very unique distribution of air holes, including quasi–periodic formats, or even asymmetrical formats [9], which can increase the rate of precision and speed in the SPR response. In this case, the MOF-based SPR presented in this paper, can be easily constructed, as it presents a smaller number of distributed holes, better organized in the transversal section. Thus, it is quite clear that an SPR can be compatible with several models of existing optical structures, as is the case of MOF.

Microstructured optical fibers are structures that have holes, usually of air, along their entire direction of propagation. The function of these holes, for the most part, is to create photonic bandgaps, causing the signal to be confined in a certain region of the fiber. As such, these holes can have different shapes, such as rectangular and elliptical [7,8], for example, in addition to being able to cause multicores in the fiber [10,11]. The applications, in which the MOF appear are the most varied, such as for chromatic dispersion adjustment [12], interferometers operating point adjustment [13], imaging [14] and even gas sensing, using the holes of the structure as microchannels [15].

When studying SPR-based MOF sensors, the effect that the holes cause on the sensor is highly relevant to obtain the desired results. The air holes can be utilized as guides for the internal fields of fiber, producing LSPR in the desired regions, which is where the analysis materials are. Therefore, parameters such as sensitivity, range of operation, confinement losses, among others, are directly determined by the distribution, shape and parameterization of the MOF holes.

It is important to note that the study of MOF holes are of great importance when it is desired to build SPR-based sensors, as they provide a wide variety of devices [7,8,9,10,11,12,13,14,15,16,17,18,19,20,21,22,23,24,25,26,27,28,29,30,31,32], each unique and with distinct properties, which can be used in specific applications.

In this work, the analysis of an SPR-based optical sensor on a substrate formed by a MOF with a D-shape is proposed. In the following sections are presented the simulation theories, the proposed sensor design, the results of the sensor characteristics that were obtained in the development of this work, as well as a comparison of the proposed sensor with other studies found in the literature, and finally, the conclusions and possible applications of this device.

## 2. Plasmonic Sensor Design and Simulation

**Figure 1:**
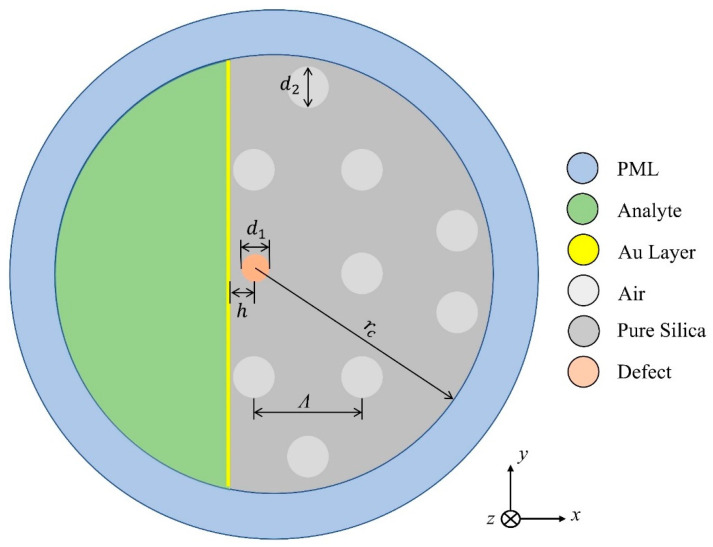
Cross section sensor design.

In this work, a D-shaped fiber was developed as a sensor. Normally, these fiber models are not easy to theoretically model, mainly due to the disturbance in its axial symmetry, when one of its edges is subjected to a polishing process. For the MOF project in question, polishing occurs in its longitudinal plane, by removing a part of the fiber ${\mathrm{SiO}}_{2}$ core. The removal of a part of the core allows the evanescent field, which propagates in this region of the optical fiber, to be coupled to the external environment. To take advantage of this phenomenon, a thin film of gold is fixed to the ${\mathrm{SiO}}_{2}$ core, where part of the material was removed, where on the other side of this blade is placed the material that constitutes the analyte, as seen in Figure 1, which shows the design of the structure used as an optical sensor. Thus, a change in the coupling condition is caused when the structure is excited at optical frequencies.

In Figure 1, $r_{c}=17\;\mu m$, represents the radius of the ${\mathrm{SiO}}_{2}$ core; $d_{1}=1.6\;\mu m$, represents the diameter of the defect, included in the center of the structure; $d_{2}=2.8\;\mu m$ represents the diameter of the air holes, quasi-periodically distributed; Λ=7.93 μm, is the distance between the centers of the air holes (pitch). In addition, in this work the thickness of the gold layer used was $t_{\mathrm{Au}}=50\;\mathrm{nm}$, while the distance from the center of the structure to the gold film used was d=2.55 μm. For surface wave absorption, a circular type PML (Perfectly Matched Layer), of thickness $t_{\mathrm{PML}}=1\;\mu m$, was used.

For the theoretical analysis of the structure, a formulation [33] based on the finite element method (FEM) is used. For the purpose of applying the FEM, the structure was discretized in 12,171 triangular elements, concentrating smaller elements, with minimum size of 10.8 nm, on the Au film. In the other regions of the structure, the maximum size of the elements was 4.68 μm. The x and y coordinates represent the transverse directions and z represents the direction of propagation, as shown in Figure 1. In this case, the formulation is directly obtained from the Maxwell equations, reaching a global matrix equation given by: $\left[A\right]\left\{\varphi \right\}=n_{\mathrm{eff}}^{2}\left[B\right]\left\{\varphi \right\}$. Here [A] and [B] are complex and sparse matrices. In addition, this equation can be solved using an iterative subspace method, where refractive index (RI) doping variations are included directly in the calculations. Since, $n_{\mathrm{eff}}$ represents the effective refractive index, it can be directly obtained through the matrix expression mentioned above.

Other important aspects for the good performance of the sensor are the materials that form the signal guiding structure, as well as the plasmonic response of the gold film when subjected to optical frequencies. As the structure is composed of materials with different permittivity and when these materials are excited at optical frequencies, an effective permittivity is obtained, from which the effective refractive index of the structure can be calculated.

In this design, the guiding structure is composed of a MOF with a ${\mathrm{SiO}}_{2}$ core, in which a defect is introduced, as shown in Figure 1. The permittivity of the material that forms the defect is initially constituted by ${\mathrm{SiO}}_{2}$, and then tests are carried out using doping with Germanium Dioxide $\left({\mathrm{GeO}}_{2}\right)$. The concentrations of ${\mathrm{GeO}}_{2}$ used were 4.1%, 6.3%, 13.5% and 19.3%, respectively. These doping values were chosen due to implications for the construction process. It is important to note that a small percentage of atoms of the dopant material in the silica crystal lattice may produce drastic changes in its dielectric proprieties, in this case, the doping percentages were selected so as not to cause structural damage in the fiber manufacturing process. In conventional fibers, the maximum percentage of core varies is around 4% so that no damage occurs when pulling the fiber. On the other hand, in microstructured optical fibers (MOF) the pure silica matrix supports higher percentages of ${\mathrm{GeO}}_{2}$, and in this case, these values were chosen to meet the manufacturing needs.

The air holes of MOF are distributed in a quasi-periodic way in the cross section of D-shaped fiber and extend along the direction of propagation (z). The geometric distribution of the holes, as well as their respective dimensions, were obtained based on [12] using genetic algorithms with fitness function guided by a local search space, where the optimized parameters were the radius of the air holes, the radius of the defect and the distance between the centers of the air holes. The plasmonic element used is the gold and sensing analysis is verified by the RI of the analyte ($n_{a}$).

Generally, to measure the capacity of a plasmonic sensor, several parameters can be used, however, the most common are the confinement losses (CL) and wavelength sensitivity (WS). However, parameters such as amplitude sensitivity (AS), transmission coefficient (T) and plasmonic field amplitude can be used. The effect of noise and distortions in the fiber were not considered in the simulations.

Confinement loss is a common effect, which usually is associated with air holes when the fiber is microstructured. That is, it can be directly related to the size, distribution, number of air holes of the MOF and the wavelength of operation [34]. The CL can be obtained according to Equation (1):

(1) CL(dB/cm)=8.686×2×π×104×Im(neff)λ 

where $n_{\mathrm{eff}}$ is the complex effective refractive index, obtained from the modal analysis of the structure and λ is the wavelength of operation in micrometers.

The wavelength sensitivity, or spectral sensitivity, represents the rate of variation in the excitation wavelength in relation to $n_{a}$, that is, the variation in the analyte will be detected by the change of peak resonance [27,28,35], and its result defines WS in terms of the refractive index unit (RIU). The WS can be obtained by Equation (2):

(2) $$\mathrm{WS}=\frac{∆\lambda }{\Delta n}\;\left(\mathrm{nm}/\mathrm{RIU}\right)\;,$$

where ∆λ is the variation in the wavelength of the peak resonance and Δn indicates the variation in the refractive index.

In all simulations, to obtain the permittivity of the materials, the Sellmeier equation [35] was used, according to Equation (3):

(3) $$\varepsilon \left(\lambda \right)=1+\overset{3}{\underset{k=1}{\sum }}\frac{B_{k}\lambda^{2}}{\lambda^{2}-C_{k}^{2}},$$

where λ represents the excitation wavelength in μm, B and C are the coefficients of the Sellmeier equation that vary according to the material used. Table 1 shows the values of Sellmeier coefficients used in this work.

In addition, Table 2 shows the refractive index values obtained for the different silica doping with ${\mathrm{GeO}}_{2}$, with the direct application of the Sellmeier equation, presented in Equation (3). As the Sellmeier equation returns a result as a function of the wavelength, then for the case of this article, a step of 0.5 μm in the wavelength was considered and in this way the RI values for the entire analysis spectrum were obtained.

It is important to highlight that the percentages of doping could not be optimized as they were obtained from experimental studies [35] on Sellmeier equations, so the coefficients are predetermined.

**Figure 2:**
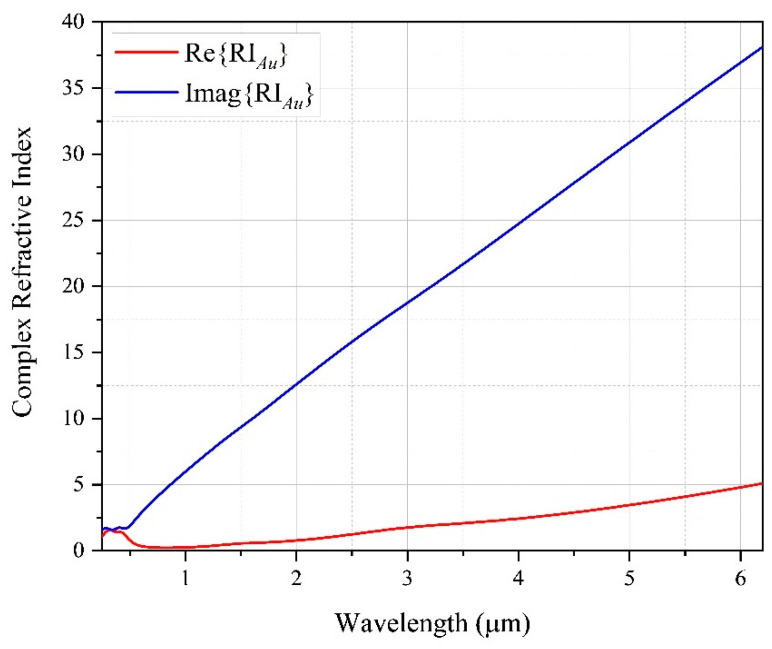
Real and imaginary part of the gold RI, for the wavelength range between 0.24797 μm and 6.1992 μm.

Due to the formulation used in this work to perform the simulations, all materials must be treated as dielectric. Therefore, to obtain the complex permittivity of the plasmonic element used in this work, the Drude–Lorentz model [36] was applied. This model represents a widespread way of determining the complex permittivity of metallic materials as a function of wavelength. Thus, the refractive index of the plasmonic element can be obtained directly by application of the Drude–Lorentz model. Figure 2 shows the variation in the real part (red line) and the imaginary part (blue line) of the Au refractive index.

## 3. Results and Discussions

To perform the simulations, a proper formulation was used [33]. The formulation uses the Helmholtz wave equation, obtained from the Maxwell Equations, considering the complex permittivity of dielectric material with transverse anisotropy. In this algorithm, the wave equation is numerically solved using the FEM in conjunction with the Galerkin Method. The cross section of the structure is discretized with triangular elements and the characteristics of the materials used are directly introduced into the permittivity (including the gold layer). To limit the computational domain, Perfectly Matched Layers (PML) of the circular type are applied directly in the formulation. The computational code is implemented in the FORTRAN language and the results are exported to be plotted in other numeric computing platforms. In addition, to generate the mesh of the structure a mesh generation software is used, and the data is generated on these platforms and exported directly to the computation algorithm.

**Figure 3:**
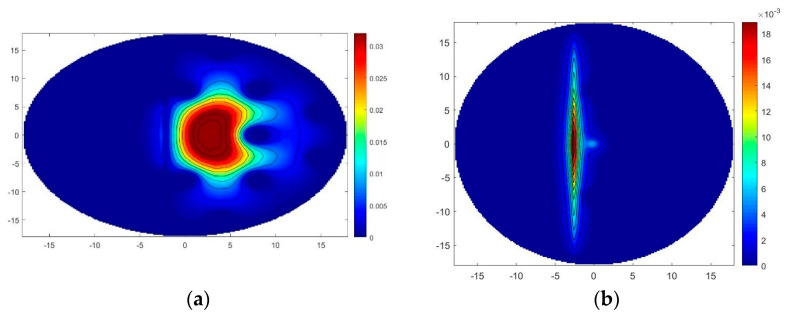
Magnetic field in fiber for: (**a**) Fiber with ${\mathrm{SiO}}_{2}$ defect, $n_{a}=1.43$ and λ=1.55 μm; (**b**) Defect with doping ${\mathrm{SiO}}_{2}+{\mathrm{GeO}}_{2}\left(13.5\%\right),{\;n}_{a}=1.43$ and λ=1.55 μm.

First, the modal analysis of the structure was performed to obtain the fundamental mode, or first order mode, as well as the verification of the emergence of LSPR, through the contour lines of the magnetic field (**H**), polarized in the y direction, presented in Figure 3.

**Figure 4:**
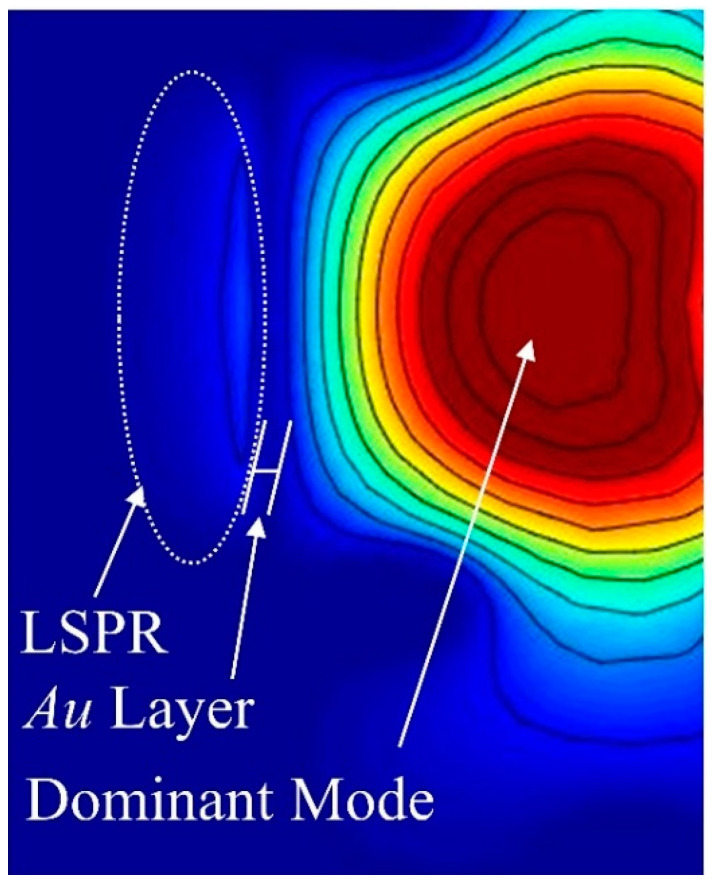
Detail of the LSPR in fiber with ${\mathrm{SiO}}_{2}$ defect.

Figure 3a shows that the energy concentration of the fundamental mode in the core is contained by the air holes of the MOF, however, it is perceived as the appearance of LSPR, as can be analyzed in more detail in Figure 4.

In Figure 3b, due to the percentage of doping added in the defect, there is a greater passage of energy from the fundamental mode to the plasmonic mode. This is explained by the increase in energy within the defect, being closer to the metal-dielectric interface, and when the structure reaches the plasmonic frequency, the intensity of the plasmonic mode becomes consequently higher.

Analyzing Figure 4, the localized appearance of surface plasmons is confirmed. It is also confirmed that the field inside the region of the gold layer is practically null, which in fact proves the effect of SPR.

For the defects doped with ${\mathrm{GeO}}_{2}\;\left(4.1\%\right)$, ${\mathrm{GeO}}_{2}\;\left(6.3\%\right)$ and ${\mathrm{GeO}}_{2}\left(19.3\%\right)$ the same effect is observed, however, what occurs are variations in the intensities of the fundamental and plasmonic modes, which will directly interfere in the parameters of the confinement losses and wavelength sensitivity.

In this work, four variations in doping in the defect immersed in the MOF core will be analyzed and compared, in addition to the analysis considering doping of 0%, which corresponds to pure silica.

The visualization of the plasmonic and fundamental mode, can be observed more easily from the analysis of the one-dimensional electric field (**E**) component, calculated from a cross-sectional line positioned in y = 0. Thus, one can see in a more simplified way the effects that occur in plasmonic and fundamental modes, in relation to the variations in $n_{a}$.

**Figure 5:**
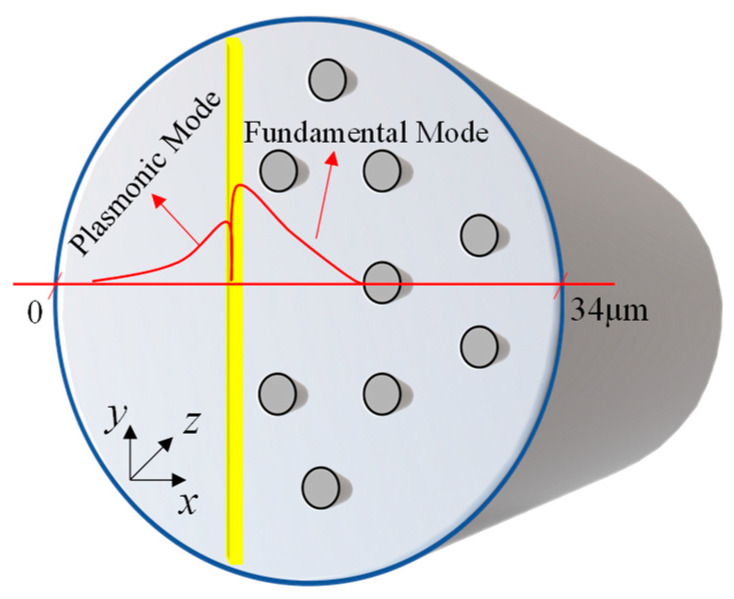
1D analysis scheme of plasmonic and fundamental modes.

Figure 5 presents a horizontal cut performed in the center of the fiber, to show a generic example of the one-dimensional **E**-field, in which the plasmonic modes and the fundamental modes are formed. All simulations used to analyze the electric field were performed for the excitation wavelength of 1.55 μm. This wavelength was chosen due to the greater number of applications with optical fibers being around this wavelength range, however, it should be noted that the sensor was designed to operate in a wider spectrum.

**Figure 6:**
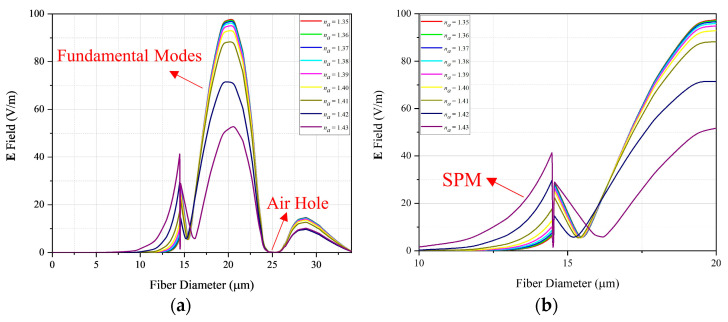
One-dimensional **E**-field for sensor with ${\mathrm{SiO}}_{2}$ defect for: (**a**) Entire diameter; (**b**) Close to SPR.

Figure 6 shows one-dimensional **E** curves, considering the sensor with ${\mathrm{SiO}}_{2}$ core without the defect in the center of the structure, for a wavelength of 1.55 μm.

In Figure 6a, it can be observed that for analytes within the RI range of 1.35 to 1.43, the effect of LSPR occurs, and a portion of the energy contained in the fundamental mode is coupled to the plasmonic mode, that is, as $n_{a}$ increases, the energy of the fundamental mode decreases, while that of the plasmonic mode increases. For $n_{a}=1.35$, the fundamental mode reaches a maximum of 97.83 V/m, while the maximum plasmonic mode is 14.46 V/m. At the other extreme, for $n_{a}=1.43$, the peak of the fundamental mode is 53.36 V/m and that of the plasmonic mode is 41.62 V/m, about 45% of the fundamental mode. In addition, the detection range of this sensor configuration operates for analytes with RI between 1.35 and 1.42. Figure 6a also shows a bandgap where the air hole of the MOF is located, which is already an expected effect of these type of fibers, there is also a discontinuity of the **E**-field in the gold laminate, which was also predictable, since due to the skin effect, the field inside a conductive material tends to be null.

Figure 6b presents a local analysis of the **E**-field, around the region of the gold layer, which is the region where the surface plasmons will appear. Figure 6b confirms that as the energy concentrated in the fiber defect decreases, the energy located in the plasmonic mode increases.

**Figure 7:**
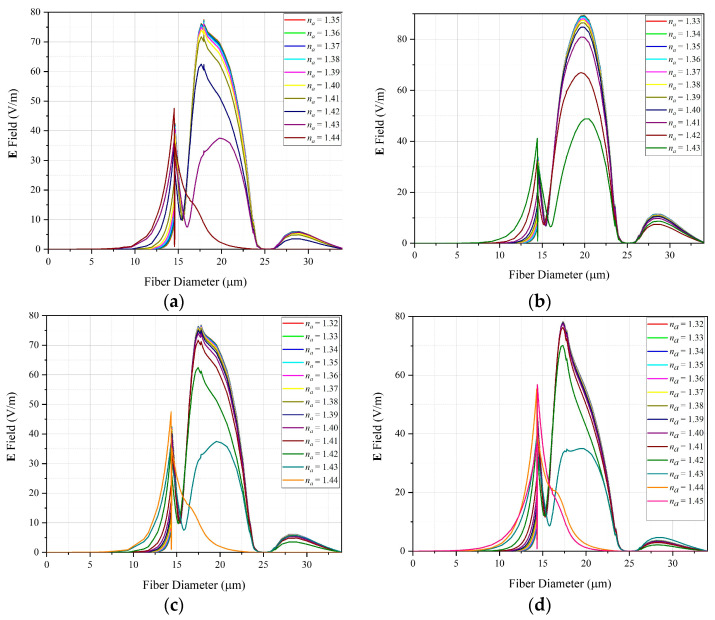
One-dimensional **E**-field for sensor with defect doped with ${\mathrm{GeO}}_{2}$: (**a**) 4.1%; (**b**) 6.3; (**c**) 13.5%; (**d**) 19.3%.

Figure 7 shows the variation in **E**-field, considering the introduction, in the core of the MOF, of a defect filled with silica material doped with germanium. In this case, a similar behavior is observed, as can be seen in the following curves. For simulation purposes, graphs in Figure 6 and Figure 7 were generated with the same number of points.

In Figure 7, it is observed that with the introduction of the ${\mathrm{GeO}}_{2}$ in the defect, there is an increase in the detection range of the proposed sensor. Figure 7a, shows results considering the material of the defect doping with ${\mathrm{GeO}}_{2}\;\left(4.1\%\right)$. Here, the operating range of analyte for RI occurs for values from 1.35 to 1.44. With $n_{a}=1.34$ the maximum value of the fundamental mode is 77.46 V/m, while that of the plasmonic mode is 8.67 V/m, for $n_{a}=1.44$, the highest value of the fundamental mode is 16.5 V/m while that of plasmonic mode is 47.76 V/m. Figure 7b, shows results for a defect doping with ${\mathrm{GeO}}_{2}\;\left(6.3\%\right)$, this configuration allows the sensor to operate for analytes with RI ranging from 1.33 to 1.43, so for $n_{a}=1.33$ the maximum value of the fundamental mode is 89.66 V/m while the plasmonic mode is 6.03 V/m, for $n_{a}=1.43$, the maximum value of the fundamental mode is as 41.07 V/m while that of the plasmonic mode is 48.82 V/m. In Figure 7c, the doping in defect with ${\mathrm{GeO}}_{2}\;$was 13.5% for a detection range of 0.12 RIU, with $n_{a}$ varying from 1.32 to 1.44. Para $n_{a}=1.32$ the maximum value of the fundamental mode is as 76.98 V/m while that of the plasmonic mode is 7.13 V/m, for $n_{a}=1.44$ the peak value of the fundamental mode is as 16.11 V/m while the plasmonic mode is 47.69 V/m. Finally, in Figure 7d, the percentage of doping with ${\mathrm{GeO}}_{2}$ was 19.3%. In this case, the highest detection range was observed among the cases studied (0.13 RIU), which allowed a sensor operating range with $n_{a}$ varying from 1.32 to 1.45. For $n_{a}=1.32$, the maximum value of the fundamental mode is 78.5 V/m while that of the plasmonic mode is 7.49 V/m, for the $n_{a}=1.44$, the maximum value of the fundamental mode is as 19.37 V/m and that of the plasmonic mode is 36.44 V/m.

According to the results presented in Figure 7, it is noticed that the introduction of the defect in the core of the MOF, with material consisting of ${\mathrm{SiO}}_{2}\;$doped with ${\mathrm{GeO}}_{2},$ causes an increase in the operating range of sensors based on SPR. This is a positive aspect, as plasmonic sensors, despite having a high sensitivity, are also known to have a narrow range of operation, which can greatly limit their applications.

**Figure 8:**
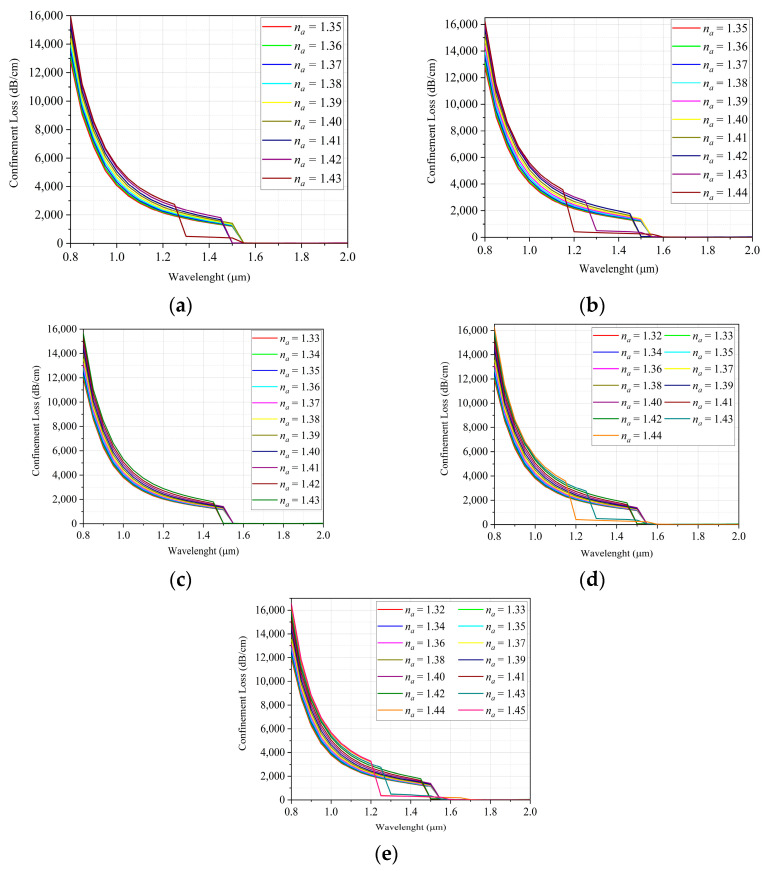
Confinement loss versus wavelength, considering the defect: (**a**) without doping; (**b**) doped with ${\mathrm{GeO}}_{2\;}\left(4.1\%\right)$; (**c**) doped with ${\mathrm{GeO}}_{2}\;(6.3\%$ ); (**d**) doped with ${\mathrm{GeO}}_{2}\;\left(13.5\%\right)$; (**e**) doped with ${\mathrm{GeO}}_{2}\;\left(19.3\%\right)$.

The result of the CL and WS analysis of the sensor is presented below to verify the effect of the defect introduction in these parameters, considering the values of the $n_{a}\;$ranges obtained in the projects. Figure 8 investigates the behavior of confinement loss curves of the proposed configurations for the entire spectrum of analysis.

According to the results presented in Figure 8, it can be observed that the CL curves follow a similar pattern. It can be seen that, within an analysis range of 0.8 to 1.2 μm, the lower the $n_{a}$, the lower are the losses, and as the RI of the analyte increases, the losses also increase. Figure 8b,d show that for analytes with $n_{a}=1.44$, there were higher values of CL occurred, as expected, however, it is noticed that at the wavelength of 1.15 μm there is a rapid decrease in the values of those losses. On the other hand, in Figure 8e, the upper limit of the sensor detection range is increased to 1.45, due to the high concentration of ${\mathrm{GeO}}_{2}$, which made the curves more stable, for this operating limit. It can be seen that in all situations around the wavelength of 1.55 μm, the confinement losses are strongly reduced, stabilizing at values between 15 and 60 dB/cm.

**Figure 9:**
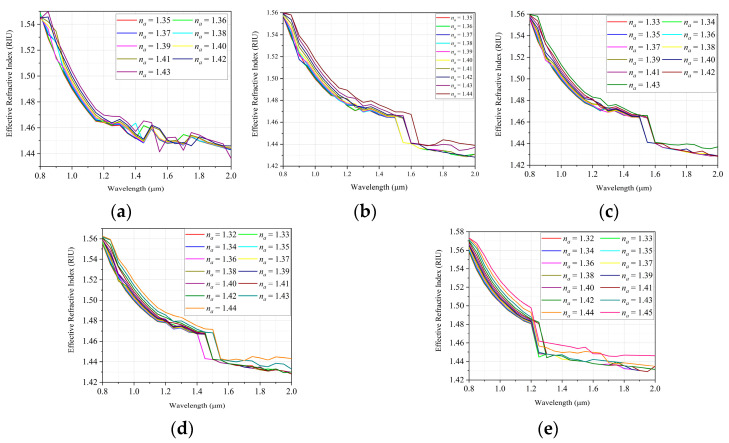
Relationship between effective RI and wavelength for the various detection ranges: (**a**) Defect without doping; (**b**) Doping with ${\mathrm{GeO}}_{2}\;\left(4.1\%\right)$; (**c**) Doping with ${\mathrm{GeO}}_{2\;}\left(6.3\%\right)$; (**d**) Doping with ${\mathrm{GeO}}_{2}\;\left(13.5\%\right)$; (**e**) Doping with ${\mathrm{GeO}}_{2}\;\left(19.3\%\right)$.

Figure 9 shows the effective RI variation as a function of wavelength for different RI values of the analyte. These results are necessary to obtain the wavelength sensitivity as a function of the effective RI value of the analyte.

**Figure 10:**
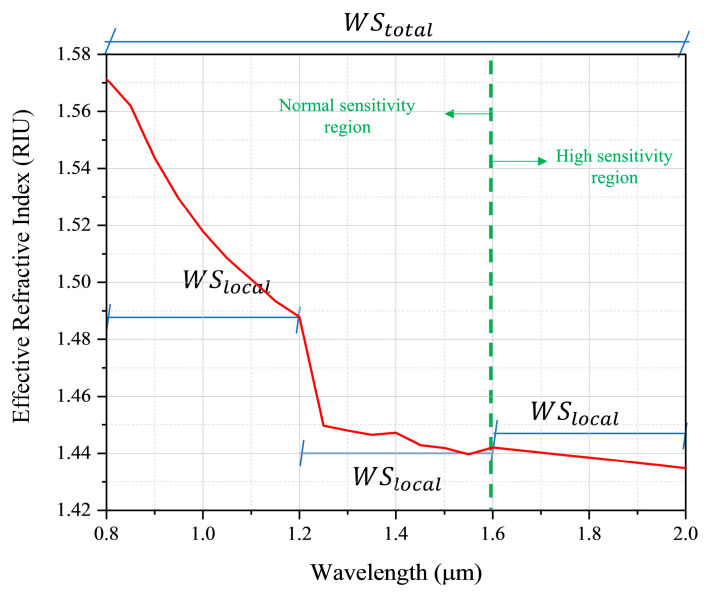
Sensor sensitivity regions scheme for doping with ${\mathrm{GeO}}_{2}\;\left(19.3\%\right)$ and $n_{a}=1.41$.

From the variation in the wavelength, in relation to the effective refractive index, it is possible to obtain the spectral sensitivity of the proposed sensor. The sensitivity can be obtained in full range, taking into account the entire spectrum, however, it can also be obtained locally, taking into account only specific intervals of wavelengths, as shown in Figure 10.

In addition, Table 3 presents the values of general and local sensitivity of the sensors, based on the results obtained in Figure 9.

The maximum WS was obtained on the sensor without any doping, for $n_{a}=1.42$, reaching the value of 12,133.47 nm/RIU, while the maximum local sensitivities were obtained in the region of 1.6 to 2.0 μm, where for the sensor without doping with $n_{a}=1.38$ the sensitivity obtained was 111,111.11 nm/RIU. For doping with ${\mathrm{GeO}}_{2}\;\left(4.1\%\right)$ and $n_{a}=1.43$ the sensitivity was 100,000.00 nm/RIU. In doping with ${\mathrm{GeO}}_{2\;}\left(6.3\%\right)$, and $n_{a}=1.43$ the obtained value was 95,238.09 nm/RIU. The device with the defect doped with ${\mathrm{GeO}}_{2\;}\left(13.5\%\right)$ obtained a maximum sensitivity with $n_{a}=1.44$, reaching the value of 235,294.12 nm/RIU. Finally, the defect doped with ${\mathrm{GeO}}_{2}\;\left(19.3\%\right)$ showed the maximum sensitivity for $n_{a}=1.45$ in the value of 190,476.19 nm/RIU, moreover, with this doping, for $n_{a}$ in the range of 1.37 to 1.45, the WS was stable at the value of 133,333.33 nm/RIU. Among all the possibilities, the total minimum sensitivity occurred in doping with ${\mathrm{GeO}}_{2}\;\left(19.3\%\right)$ and for $n_{a}=1.42$, with the value of 8658.00 nm/RIU, the minimum local sensitivity also occurred for this same concentration and RI of the analyte, in the range of 0.8 to 1.2 μm, with sensitivity of 4739.33 nm/RIU. It is noticed that the total sensitivity of the plasmonic sensor is higher when the core of the MOF is constituted only by ${\mathrm{SiO}}_{2}$.

**Figure 11:**
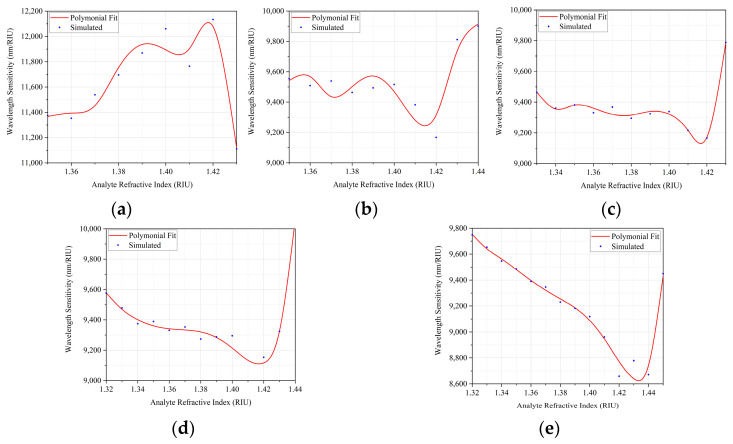
Adjustment of curves for the sensitivity values obtained: (**a**) Defect without doping; (**b**) Doping with ${\mathrm{GeO}}_{2}\;\left(4.1\%\right)$; (**c**) Doping with ${\mathrm{GeO}}_{2}\;\left(6.3\%\right)$; (**d**) Doping with ${\mathrm{GeO}}_{2}\;\left(13.5\%\right)$; (**e**) Doping with ${\mathrm{GeO}}_{2}\;\left(19.3\%\right)$.

Figure 11 shows a graph of the total sensitivity versus RI of the analytes for the various proposed dopings.

To complement Figure 11, the Table 4 presents the polynomials obtained in the curve fits performed.

Figure 11a shows the highest sensitivity, which is achieved when the effective refractive index of the structure varies from 1.41 to 1.42. For the cases, in which the defect was doped with ${\mathrm{GeO}}_{2}$, it is possible to see that when closer to the effective refractive index of 1.42, the sensitivity values are lower. It is also possible to see the case of doping with ${\mathrm{GeO}}_{2}\;\left(6.3\%\right)$, shown in Figure 11c, which showed a certain stability, where the sensitivity varied little in relation to the range of 1.35 to 1.42, referring to the effective refractive index.

Finally, Table 5 presents a comparison of the results obtained in this work with other sensors found in the literature.

## 4. Discussion

In this work, a new plasmonic sensor model was proposed using a microstructured optical fiber to detect the refractive index of reference. In addition, a study of this structure was carried out considering several ${\mathrm{GeO}}_{2}$ dopings, introduced in a circular defect of diameter d1, immersed in the center of the MOF. The sensor studied used a D-shaped optical fiber as base, in order to allow the analyte to be deposited in a polished region, located on one side of the fiber. The modes of propagation, including fundamental modes and plasmonic modes, were analyzed, as well as a detailed study into the confinement losses and wavelength sensitivity.

According to the results obtained, it was noticed that as the concentration of the dopant material located in the defect increases, the detection range of the sensor also increases, allowing the sensor to operate to detect a greater diversity of analytes. On the other hand, the highest sensitivity detected was in the structure without any doping, with a spectral sensitivity of 12,133.47 nm/RIU, and it was noticed that as the concentration of ${\mathrm{GeO}}_{2}$ increases and the sensitivity of the sensor decreases very slightly. Nevertheless, the lowest average sensitivity found was for structure with a ${\mathrm{SiO}}_{2}+{\mathrm{GeO}}_{2}\;\left(19.3\%\right)$ defect, with 9229.90 nm/RIU, which is still high sensitivity and does not prevent the application of the defect. In addition, this setting has increased the sensor detection range to operate with values between 1.32–1.45 RIU. Finally, a comparative table was presented with other structures found in the literature.

We can conclude that the proposed sensor can overcome some limitations presented in other sensors based on MOF-SPR. This is due to the fact that the vast majority of plasmonic sensors presented in the literature lose their efficiency in the detection of analytes, for a wide range of operations, and for this aspect, the device exhibits great potential for sensing applications in the biological and chemical areas.

## Figures and Tables

**Table 1:** Sellmeier coefficients.

Sensors	$B_{1}$	$B_{2}$	$B_{3}$	$C_{1}$	$C_{2}$	$C_{3}$
No doping	0.6961663	0.4079426	0.8974794	0.0684043	0.1162414	9.896161
$SiO_{2}+GeO_{2}\;\left(4.1\%\right)$	0.6867178	0.4348151	0.8956551	0.0726752	0.1151435	10.002398
$SiO_{2}+GeO_{2}\;\left(6.3\%\right)$	0.7083925	0.4203993	0.8663412	0.0853842	0.1024839	9.896175
$SiO_{2}+GeO_{2}\;\left(13.5\%\right)$	0.73454395	0.4271083	0.8210340	0.00869769	0.1119519	10.48654
$SiO_{2}+GeO_{2}\;\left(19.3\%\right)$	0.7347008	0.4461191	0.8081698	0.0764679	0.1246081	9.896203

**Table 2:** RI values obtained for different ${\mathrm{GeO}}_{2}$ concentrations.

Wavelength (μm)	No Doping	$SiO_{2}+GeO_{2}$ (4.1%)	$SiO_{2}+GeO_{2}$ (6.3%)	$SiO_{2}+GeO_{2}$ (13.5%)	$SiO_{2}+GeO_{2}$ (19.3%)
0.80	1.4533172548	1.4596845495	1.4622823847	1.4715515550	1.4810033015
**0.85**	1.4524982860	1.4588361870	1.4614292333	1.4709993384	1.4800696281
**0.90**	1.4517539550	1.4580689419	1.4606585052	1.4704900422	1.4792367010
**0.95**	1.4510651315	1.4573624396	1.4599496556	1.4700118200	1.4784802919
**1.00**	1.4504174094	1.4567013392	1.4592871988	1.4695557639	1.4777821857
1.05	1.4497997593	1.4560738981	1.4586592708	1.4691150616	1.4771284601
1.10	1.4492036097	1.4554709933	1.4580566479	1.4686844229	1.4765083153
1.15	1.4486222069	1.4548854402	1.4574720609	1.4682596813	1.4759132590
1.20	1.4480501614	1.4543115084	1.4568997094	1.4678375126	1.4753365304
1.25	1.4474831206	1.4537445737	1.4563349105	1.4674152314	1.4747726841
1.30	1.4469175294	1.4531808627	1.4557738414	1.4669906435	1.4742172864
1.35	1.4463504523	1.4526172632	1.4552133484	1.4665619352	1.4736666904
1.40	1.4457794402	1.4520511825	1.4546508033	1.4661275918	1.4731178664
1.45	1.4452024286	1.4514804385	1.4540839942	1.4656863342	1.4725682741
1.50	1.4446176596	1.4509031772	1.4535110411	1.465237071	1.4720157629
1.55	1.4440236217	1.4503178079	1.4529303310	1.4647788624	1.4714584966
1.60	1.4434190019	1.4497229527	1.4523404668	1.4643108884	1.4708948926
1.65	1.4428026489	1.4491174068	1.4517402268	1.4638324286	1.4703235752
1.70	1.4421735426	1.4485001066	1.4511285325	1.4633428423	1.4697433377
1.75	1.4415307705	1.4478701039	1.4505044226	1.4628415542	1.4691531123
1.80	1.4408735085	1.4472265460	1.4498670326	1.4623280424	1.4685519459
1.85	1.4402010046	1.4465686581	1.4492155773	1.4618018289	1.4679389798
1.90	1.4395125664	1.4458957302	1.4485493372	1.4612624715	1.4673134341
1.95	1.4388075504	1.4452071054	1.4478676464	1.4607095576	1.4666745939
2.00	1.4380853528	1.4445021703	1.4471698840	1.4601426981	1.4660217981

**Table 3:** Details of the sensitivity of the proposed sensor, for the various dopings and analytes.

Doping	$n_{a}\;(\mathrm{RIU})$	WS_Total (nm/RIU)	WS (0.8–1.2 μm) (nm/RIU)	WS (1.2–1.6 μm) (nm/RIU)	WS (1.6–2.0 μm) (nm/RIU)
No doping (0%)	1.35	11,374.41	4744.96	24,691.36	80,000.00
	1.36	11,352.89	4756.24	24,242.42	78,431.37
	1.37	11,538.46	4872.11	23,952.09	76,923.07
	1.38	11,695.90	4884.00	23,391.81	111,111.11
	1.39	11,869.43	5012.53	22,727.27	108,108.11
	1.40	12,060.30	4987.53	25,974.02	102,564.10
	1.41	11,764.70	4987.53	25,000.00	68,965.52
	1.42	12,133.47	5154.63	23,952.09	86,956.52
	1.43	11,111.11	5376.34	22,857.14	24,844.72
$SiO_{2}+GeO_{2}\;\left(4.1\%\right)$	1.35	9554.14	4901.96	11,627.90	41,666.66
	1.36	9508.72	4884.00	11,527.37	41,666.66
	1.37	9538.95	4932.18	11,396.01	41,666.66
	1.38	9463.72	4907.97	11,204.48	41,666.66
	1.39	9493.67	4968.94	11,019.28	41,666.66
	1.40	9516.26	5044.13	10,752.68	41,666.66
	1.41	9382.33	5174.64	9779.95	41,237.11
	1.42	9167.30	5095.54	10,050.25	31,746.03
	1.43	9811.94	5235.60	9546.54	100,000.00
	1.44	9900.99	5641.74	18,099.54	14,184.39
$SiO_{2}+GeO_{2}\;\left(6.3\%\right)$	1.33	9463.72	4938.27	11,695.90	34,482.75
	1.34	9360.37	4866.18	11,627.90	34,482.75
	1.35	9382.32	4901.96	11,527.37	34,482.75
	1.36	9331.25	4878.04	11,428.57	34,482.75
	1.37	9367.68	4932.18	11,299.43	34,482.75
	1.38	9295.12	4907.97	11,111.11	34,482.75
	1.39	9324.00	4968.94	10,928.96	34,482.75
	1.40	9338.52	5208.33	9975.06	34,482.75
	1.41	9216.58	5174.64	9708.73	34,188.03
	1.42	9167.30	5095.54	9950.24	32,786.88
	1.43	9787.92	5228.75	9546.53	95,238.09
$SiO_{2}+GeO_{2}\;\left(13.5\%\right)$	1.32	9577.01	5188.06	10,025.06	48,192.77
	1.33	9478.67	5115.08	9975.06	48,192.77
	1.34	9375.00	5044.13	9900.99	48,192.77
	1.35	9389.67	5069.70	9876.54	47,619.04
	1.36	9331.25	5044.13	9779.95	47,619.04
	1.37	9353.07	5089.05	9685.23	47,619.04
	1.38	9273.57	5050.50	9569.37	47,619.04
	1.39	9287.92	5108.55	9411.76	47,619.04
	1.40	9295.12	5167.95	9237.87	47,619.04
	1.41	9042.95	4889.97	9803.92	39,603.96
	1.42	9153.32	5141.39	8869.18	48,780.48
	1.43	9324.01	5263.16	8928.57	50,632.91
	1.44	10,058.67	5376.34	8583.69	235,294.12
$SiO_{2}+GeO_{2}\;\left(19.3\%\right)$	1.32	9748.17	5181.34	9324.00	133,333.33
	1.33	9654.06	5115.08	9280.74	133,333.33
	1.34	9546.53	5044.13	9216.58	133,333.33
	1.35	9486.16	5012.53	9153.31	133,333.33
	1.36	9389.67	4950.49	9090.90	133,333.33
	1.37	9345.79	4938.27	9009.00	133,333.33
	1.38	9230.76	4872.10	8908.68	133,333.33
	1.39	9181.33	4866.18	8791.20	133,333.33
	1.40	9118.54	4866.18	8620.68	133,333.33
	1.41	8961.91	4796.16	8421.05	133,333.33
	1.42	8658.00	4739.33	8368.20	62,500.00
	1.43	8778.34	4790.41	8733.62	54,054.05
	1.44	8670.52	4981.32	9280.74	26,666.66
	1.45	9448.81	5319.14	8048.28	190,476.19

**Table 4:** Comparison of performance with plasmonic sensors reported in the literature.

Figure	Polynomials
Figure 11a	$Y\left(X\right)=-6.7734\times 10^{12}+2.9314\times 10^{13}X-5.2856\times 10^{13}X^{2}+5.0825\times 10^{13}X^{3}-2.7489\times 10^{13}X^{4}+7.9285\times 10^{12}X^{5}-9.5276\times 10^{11}X^{6}$
Figure 11b	$Y\left(X\right)=-3.0959\times 10^{12}+1.3339\times 10^{13}X-2.3944\times 10^{13}X^{2}+2.2921\times 10^{13}X^{3}-1.2341\times 10^{13}X^{4}+3.5432\times 10^{12}X^{5}-4.2382\times 10^{11}X^{6}$
Figure 11c	$Y\left(X\right)=8.1793\times 10^{11}-3.5704\times 10^{12}X+6.4931\times 10^{12}X^{2}-6.2972\times 10^{12}X^{3}+3.4349\times 10^{12}X^{4}-9.9917\times 10^{11}X^{5}+1.2109\times 10^{11}X^{6}$
Figure 11d	$Y\left(X\right)=6.3266\times 10^{10}-2.7952\times 10^{11}X+5.1453\times 10^{11}X^{2}-5.0509\times 10^{11}X^{3}+2.7886\times 10^{11}X^{4}-8.2105\times 10^{10}X^{5}+1.0071\times 10^{10}X^{6}$
Figure 11e	$Y\left(X\right)=9.6532\times 10^{10}-4.2282\times 10^{11}X+7.7154\times 10^{11}X^{2}-7.5074\times 10^{11}X^{3}+4.1084\times 10^{11}X^{4}-1.1989\times 10^{11}X^{5}+1.4575\times 10^{10}X^{6}$

**Table 5:** Comparison of performance with plasmonic sensors reported in the literature.

References		Type of Sensing	RI Range	Min CL(dB/cm)	Average WS (nm/RIU)	Max WS (nm/RIU)
[8]		External	1.33–1.35	−	3558.33	4200.00
[18]		Internal	1.33–1.42	−	11,000.00	−
[27]		External	1.45–1.60	3000.00	4800.00	11,800.00
[28]		External	1.33–1.42	80.00	28,000.00	−
[29]		External	1.43–1.48	35.00	7200.00	10,000.00
[30]		External	1.33–1.39	296.00	22,000.00	−
[31]		Internal	1.33–1.38	2000.00	4600.00	7040.00
This Work	No doping	External	1.35–1.43	2100.00	11,650.63	12,133.47
	${\mathrm{SiO}}_{2}+{\mathrm{GeO}}_{2}\;\left(4.1\%\right)$	External	1.35–1.44	530.00	9533.80	9900.99
	${\mathrm{SiO}}_{2}+{\mathrm{GeO}}_{2}\;\left(6.3\%\right)$	External	1.33–1.43	1980.00	9366.80	9787.92
	${\mathrm{SiO}}_{2}+{\mathrm{GeO}}_{2}\;\left(13.5\%\right)$	External	1.32–1.44	600.00	9380.02	10,058.67
	${\mathrm{SiO}}_{2}+{\mathrm{GeO}}_{2}\;\left(19.3\%\right)$	External	1.32–1.45	2000.00	9229.90	9748.17
